# Volumetric parameters on ^18^F-FDG PET/CT predict the survival of patients with gastric cancer associated with their expression status of c-MET

**DOI:** 10.1186/s12885-019-5935-3

**Published:** 2019-08-08

**Authors:** Guobing Liu, Yan Hu, Xi Cheng, Yan Wang, Yushen Gu, Tianshu Liu, Hongcheng Shi

**Affiliations:** 1 Department of Nuclear Medicine, Zhongshan Hospital, Fudan University, No. 180 in Fenglin Road, Shanghai, 200032 People’s Republic of China; 2 Department of Medical Oncology, Zhongshan Hospital, Fudan University, No. 180 in Fenglin Road, Shanghai, 200032 People’s Republic of China

**Keywords:** Gastric cancer, Prognosis, 2-[18F] Fluoro-2-deoxy-D-glucose, ^18^F-FDG, Positron emission tomography/computed tomography (PET/CT)

## Abstract

**Background:**

This study aimed to investigate the prognostic value of volumetric parameters on ^18^F- fluoro-2-deoxy-D-glucose (^18^F-FDG) positron emission tomography/computed tomography (PET/CT) in gastric-cancer patients, according to the expression status of c-MET (MET proto-oncogene, receptor tyrosine kinase), which was previously unclear.

**Methods:**

The study included 61 patients with advanced gastric cancer. Data on the baseline ^18^F-FDG PET/CT, clinical-pathological information, progression-free survival (PFS), and overall survival (OS) were collected. The maximum standardized uptake value (SUV_max_), peak SUV (SUV_peak_), metabolic tumor volume (MTV), and total lesion glycolysis (TLG) of gastric tumors in situ were measured on PET/CT. The expression status of c-MET was recorded based on immunohistochemical staining. Associations between the parameters on PET/CT and patients’ survival outcomes were analyzed in relation to expression status of c-MET.

**Results:**

Patients with positive c-MET expression had significantly shorter PFS (11.5 vs. 17.6 months, *P* = 0.039) and OS (17.0 vs. 24.3 months, *P* = 0.043), and had gastric tumors with a larger MTV (70.8 ± 53.11 vs. 41.1 ± 52.32, *P* = 0.034) and TLG (428.39 ± 442.95 vs. 205.7 ± 354.40, *P* = 0.039), compared with those with negative c-MET expression. However, SUV_max_ (9.6 ± 7.40 vs. 8.0 ± 4.91, *P* = 0.335) and SUV_peak_ (7.7 ± 5.99 vs. 6.62 ± 4.08, *P* = 0.438) were similar between these two patient groups. In patients with c-MET-positive tumors, MTV and TLG were independent factors in predicting patient OS after correction by distant metastasis (hazards ratio = 1.014 and 1.002, respectively; *P* = 0.024 and 0.027, respectively), while these associations were not significant in patients with c-MET-negative tumors.

**Conclusions:**

Patients with c-MET-positive gastric cancer had higher MTV and TLG values compared to those with c-MET-negative gastric cancer. In patients with c-MET-positive gastric cancer, volumetric parameters on ^18^F-FDG PET/CT have prognostic value for patient overall survival.

## Background

The prognosis of patients with gastric cancer remains dismal, although various treatment modalities have advanced in recent years [1]. Clinically, 2-[18F] Fluoro-2-deoxy-D-glucose (^18^F-FDG) positron emission tomography/computed tomography (PET/CT) has been widely used in diagnosis, staging, restaging, therapy-response assessing, and prognosis predicting of patients with gastric cancers [2–4]. Especially, the volumetric parameters, like the metabolic tumor volume (MTV) and total lesion glycolysis (TLG), have shown advantages over the commonly used maximum standardized uptake value (SUV_max_) in predicting prognosis of patients with gastric cancer [3, 5, 6].

The interaction between the c-MET (MET proto-oncogene, receptor tyrosine kinase) and its ligand, hepatocyte growth factor (HGF), is commonly related to tumorigenesis. Overexpression of c-MET has been reported as associated with poor prognosis in patients with gastric cancer [7–9]. Treatment targeting c-Met is a promising therapeutic approach for patients with c-MET-amplified gastric cancer. Indeed, phase III trials of onartuzumab and rilotumumab treatment of patients with gastric cancer overexpressing c-MET and a considerable number of phase I and phase II trials of agents targeting c-MET are ongoing [10–12]. Therefore, it is necessary to develop a method that can indicate c-MET expression to stratify patients. Until now, no study has investigated the correlation between FDG uptake and c-MET expression in gastric cancer.

In the present study, we attempted to investigate the prognostic value of semi-quantitative parameters on ^18^F-FDG PET/CT in patients with advanced gastric cancer undergoing chemotherapy. In addition, the associations between these parameters and c-MET positivity of gastric tumor in situ were also analyzed.

## Methods

This study was approved by the Ethics Committee of Zhongshan Hospital of Fudan University (approval number: IRB2015–098) and was performed in accordance with the Helsinki Declaration of 1975, as revised in 2000. Written informed consent was obtained from all patients.

### Study patients

This study included and followed up 61 patients with advanced gastric cancer, who received various chemotherapies (including neoadjuvant chemotherapy, adjuvant chemotherapy, and palliative chemotherapy) between June 2011 and October 2016 in our hospital. The inclusion criteria were as follows: 1) All patients had pathologically confirmed gastric adenocarcinoma; 2) initial clinical staging reached advanced gastric cancer, which was inoperable; 3) the status of c-MET expression could be obtained using immunohistochemical staining; 4) all patients had received ^18^F-FDG PET/CT before chemotherapy. Patients with any of the following conditions were excluded: 1) They had a second primary malignant tumor; or 2) they had another death-threating illness. Clinicopathological information, including sex, age at diagnosis, pathological differentiation, Lauren classification, and baseline serum concentrations of carcinoembryonic antigen (CEA), cancer antigen (CA) 19–9, and CA 72–4, were documented.

### PET/CT acquisition

All patients fasted for about 6 h before PET/CT examination. Then, patients were injected with ^18^F-FDG (3.7 MBq/kg). The mean dose was 295.1 (range, 238.2–476.3) MBq. About one hour (mean ± standard deviation [SD], 63.1 ± 12.5 min) later, patients were suggested to drink water as much as possible, right after which PET/CT scanning were performed using a hybrid GE Discovery VCT 64 PET/CT scanner (General Electric, Milwaukee, WI, USA) from the proximal thigh to the skull base. Non-enhanced helical CT scanning was performed initially (200 mA, 120 kV, matrix 512 × 512, 0.8 s per rotation), and the CT images were reconstructed to a slice thickness of 1.5 mm for review. Thereafter, PET acquisition was performed (2 min/bed position) in three-dimensional mode, and images were reconstructed using ordered-subsets expectation maximization iterative reconstruction.

### Imaging analysis

**Fig. 1:**
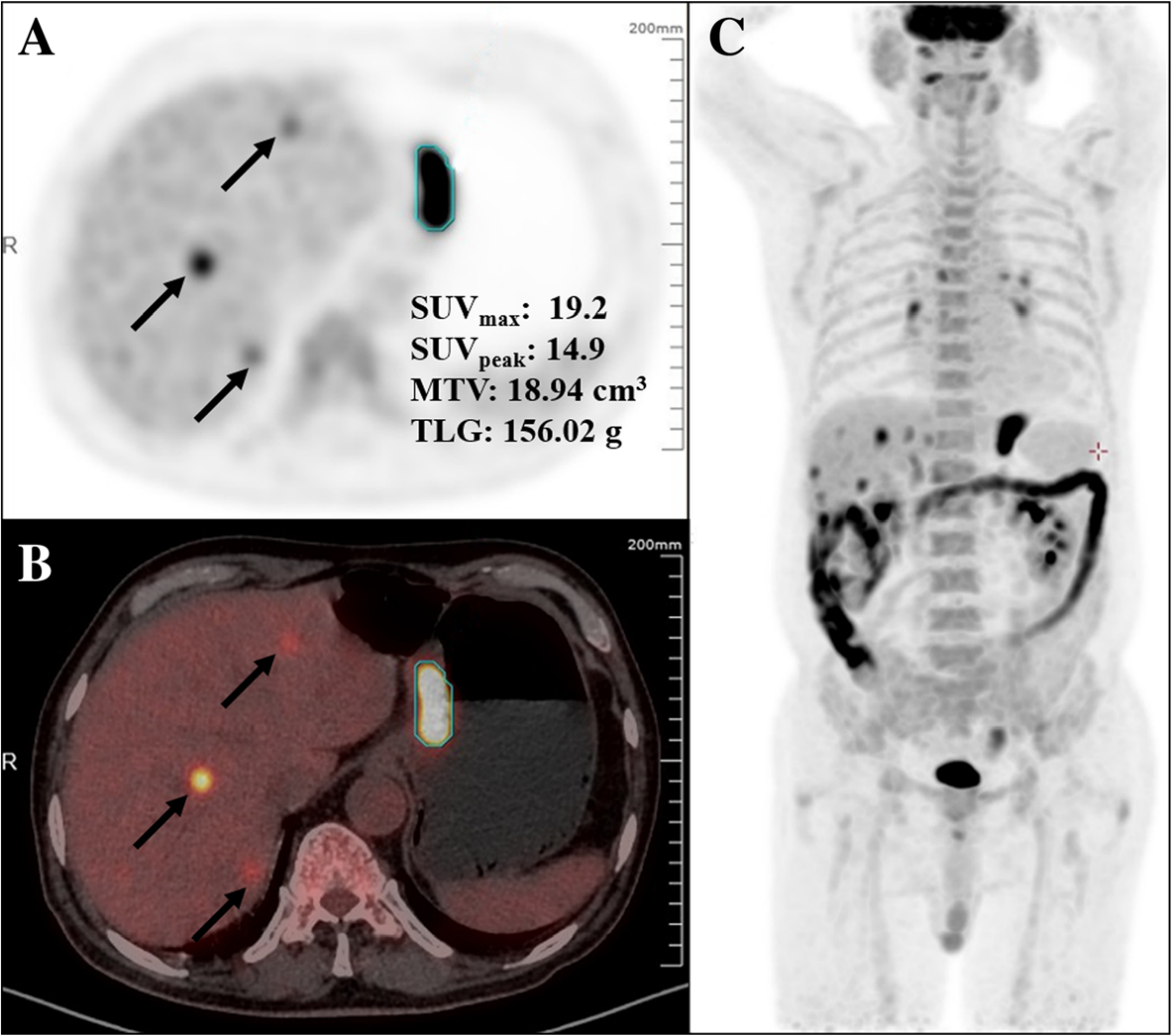
PET/CT images illustrating tumor volume delineation of a typical gastric cancer. **a**, transverse PET image; **b**, transverse PET/CT fusion image; **c**, maximum intensity projection image. Circles in **a** and **b** denote volume of interest under a threshold of SUV ≥ 2.5. In addition, multiple metastases can be visualized in the liver (arrows). PET, positron emission tomography; CT, computed tomography; SUV_max_, maximum standardized uptake value; SUV_peak_, peak standardized uptake value; MTV, metabolic tumor volume; TLG, total lesion glycolysis

Two physicians (A, G.L.; B, Y.H.), who each had worked for more than 3 years in department of nuclear medicine, evaluated the PET/CT images and measured the semi-quantitative parameters independently. First, patients were classified into two groups: Those with locoregional malignancy (including regional lymphadenopathy) and those with regional malignancy with distant metastasis, based on the PET/CT findings. Then, volumetric parameters of the tumors in situ were measured on an uWS-MI R001 workstation (United Imaging, Shanghai, China), according to the method described in a previous study [5]. The isocontour method was used to create volume of interest (VOI) around the tumor. For lesions with SUV_max_ > 2.5, a threshold of SUV ≥ 2.5 was used; however, for lesions with SUV_max_ ≤ 2.5, a 40% SUV_max_ threshold was used (Fig. 1). Each VOI generated a SUV_max_, a peak SUV (SUV_peak_), TLG (g), and MTV (mL). SUV_max_ was defined as the maximum SUV from a single pixel anywhere within the VOI, while SUV_peak_ was the highest mean SUV from a fixed 1-cm^3^ spherical VOI centered over the highest metabolic part of the tumor. MTV was defined as the sum of the metabolic volume above the predefined threshold, while TLG was defined as the product of the MTV and the mean SUV in the VOI. Observer A performed the measurements twice at an interval of over two months for testing reproducibility.

### Pathological classification and c-MET expression assay

Gastroscopy sampling during the initial diagnosis was used to evaluate tumor pathology and c-MET expression. Pathologically, the tumors were classified into well differentiated adenocarcinoma (WDA, including papillary adenocarcinoma and tubular adenocarcinoma), moderately differentiated adenocarcinoma (MDA), poorly differentiated adenocarcinoma (PDA), signet-ring cell carcinoma (SRC), and mucinous adenocarcinoma (MAC), according to the Japanese classification of gastric cancer [13]. In addition, the tumors were classified into two microscopic growth types on the basis of the Lauren classification: Intestinal and non-intestinal. Diffuse, mixed, and unclassifiable types were included in non-intestinal type. Immunohistochemical staining for c-MET was performed according to the institutional standard process. According to a previous method [14, 15], the intensity of the membrane staining for c-MET was scored as follows: Score 0, no reactivity or less than 50% of tumor cells with any membranous reactivity; score 1+, 50% or more of tumor cells with weak membranous reactivity but less than 50% with moderate or higher membranous reactivity; score 2+, 50% or more of tumor cells with moderate or higher membranous reactivity but less than 50% with strong membranous reactivity; and score 3+, 50% or more of tumor cells with strong membranous reactivity. Membrane staining intensity with score of 2+ or 3+ was defined as c-MET positive.

### Treatment and follow up

Of the 61 included patients, 51 received neoadjuvant chemotherapy, then radical surgery, and adjuvant chemotherapy after surgery. The other 10 patients only received palliative chemotherapy. The chemotherapy regimen was one of the following: oxaliplatin and capecitabine; epirubicin, oxaliplatin and capecitabine; docetaxel, oxaliplatin, and 5-FU; oxaliplatin and 5-FU; docetaxel and oxaliplatin. According to the National Comprehensive Cancer Network guidelines [16], all patients were followed up, including history taking, physical examination, serum tumor marker testing, abdominopelvic CT or magnetic resonance imaging (MRI) scanning, and gastroscopy. Patients were assessed every 3–4 months in the first 3 years and every 4–6 months, thereafter. Overall survival (OS) was the primary endpoint, which was defined as the time (in months) from the date of the baseline PET/CT scanning to the date when patients died from any cause. The secondary endpoint was progression-free survival (PFS), which was defined as the time (in months) from the date of the baseline PET/CT scanning to the date of confirmed progression. Progression was defined as at least a 20% increase in the sum of the diameters of lesions, according to the response evaluation criteria in solid tumor (RECIST 1.1). If progression was suspected, additional pathological diagnosis was performed to confirm it. Patients last known to be alive were censored at the date of last contact.

### Statistical analysis

Inter-observer variabilities in the measurements on PET/CT between observer A and B, and intra-observer variabilities between the two measurement times performed by observer A were evaluated by calculating intraclass correlation coefficients (ICCs). An ICC greater than 0.75 indicated good agreement. Mean values recorded by the two observers were used for the final analysis. Clinicopathological characteristics of the patients and the semi-quantitative measurements on ^18^F-FDG PET/CT were compared between the c-MET positive and negative patients. Comparisons of categorical variables were performed using the chi-squared test and Fisher’s exact test if there was any cell of the crosstab (a table indicating the relationship between two or more variables) that had an expected count less than five. Comparisons of continuous variables were conducted using Student’s t test when a normal distribution was obtained using the Kolmogorov–Smirnov test, otherwise the Mann–Whitney U test was used. Kaplan–Meier survival analysis using the log-rank test was performed to compare PFS and OS between patient groups. The univariate and multivariate Cox proportional hazard regression were used to analyze the predictive values of the clinicopathological factors and PET parameters. All statistical analyses were performed on the statistical software SPSS 20 (IBM Corp., Armonk, NY, USA), with a two-sided *p* value less than 0.05 indicating a significance.

## Results

### Clinicopathological information

Of the 61 patients included, 41 (67.2%) were men and 20 (32.8%) were women, with a median age of 62 (range, 36–81) years. There were 33 (54.1%) patients with c-MET positive gastric cancers, while the tumors in 28 (45.9%) patients were c-MET negative. Comparisons of the clinicopathological information and findings on PET/CT are summarized in Table 1. The distributions of patients’ gender; age; baseline serum concentrations of CEA, CA19–9, and CA72–4; pathological type; and Lauren classification of the tumors in situ were not different between the c-MET-positive and negative patients, with *P* values all > 0.05. Distant metastasis was also not associated with c-MET expression (*P* = 0.111). However, patients with c-MET-positive cancers had significantly shorter PFS (median in months: 11.5 vs. 17.6, *P* = 0.039) and OS (median in months: 17.0 vs. 24.3, *P* = 0.043) compared with those of patients with c-MET negative cancers.

**Table 1:** Comparisons of clinicopathological variables and semi-quantitative parameters on ^18^F-FDG PET/CT between c-Met positive and negative patients

Variables	Number (*n* = 61)	c-Met expression		*P*-values
		Positive	Negative	
Gender				
Male	41 (67.2%)	20 (60.6%)	21 (75.0%)	0.233^†^
Female	20 (32.8%)	13 (39.4%)	7 (25.0%)	
Median age (range)	62 (36–81)	61 (36–74)	64 (38–81)	0.541^#^
Pathological types				
WDA	17 (27.9%)	12 (36.4%)	5 (17.9%)	0.373†
MDA	18 (29.5%)	9 (27.3%)	9 (32.1%)	
PDA	21 (34.4%)	9 (27.3%)	12 (42.9%)	
MAC/SRC	5 (8.2%)	3 (9.1%)	2 (7.1%)	
Lauren classifications				
Intestinal	23 (46.9%)	13 (48.1%)	10 (45.5%)	0.851†
Non-intestinal	26 (53.1%)	14 (51.9%)	12 (54.5%)	
Baseline CEA (ng/ml)				
Median (range)	2.9 (0.4–776.4)	2.9 (0.4–776.4)	3.1 (1.1–242.0)	0.399^#^
Baseline CA19–9 (U/ml)				
Median (range)	9.0 (0.6–597.4)	8.4 (0.6–597.4)	10.1 (0.6–69.2)	0.510^#^
Baseline CA72–4 (U/ml)				
Median (range)	2.8 (0.8–131.2)	3.2 (0.8–131.2)	2.0 (0.9–79.3)	0.288^#^
Findings on PET/CT				
Locoregional	26 (42.6%)	11 (33.3%)	15 (53.6%)	0.111^†^
Distant metastasis	35 (57.4%)	22 (66.7%)	13 (46.4%)	
SUV_max_ (mean ± SD)	8.8 ± 6.40	9.6 ± 7.40	8.0 ± 4.91	0.335^*^
SUV_peak_ (mean ± SD)	7.2 ± 5.22	7.7 ± 5.99	6.6 ± 4.08	0.438^*^
MTV (mL, mean ± SD)	57.4 ± 54.39	70.8 ± 53.11	41.1 ± 52.32	0.034^*^
TLG (g, mean ± SD)	328.2 ± 417.44	428.4 ± 442.95	205.7 ± 354.40	0.039^*^
PFS, Median (95%CI)	14.5 (2.0–82.2)	11.5 (7.8–15.2)	17.6 (15.1–20.1)	0.039^‡^
OS, Median (95%CI)	20.0 (5–86.2)	17.0 (13.9–20.5)	24.3 (17.0–31.6)	0.043^‡^

Note. *SD* standard deviation, *CA19–9* carbohydrate antigen 19–9, *CA72–4* carbohydrate antigen 72–4, *CEA* carcinoembryonic antigen, *WDA* well differentiated adenocarcinoma, *MDA* moderate differentiated adenocarcinoma, *PDA* poorly differentiated adenocarcinoma, *SRC* signet-ring cell carcinoma, *MAC* mucinous adenocarcinoma, *PET/CT* positron emission tomography/computed tomography, *SUV* standardized uptake value, *Max* maximum, *MTV* metabolic tumor volume, *TLG* total lesion glycolysis, *PFS* progression free survival, *OS* overall survival. *, Student’s t test; †, Chi square test; ‡, Kaplan-Meier analysis with the log-rank test; #, Mann-Whitney U test

### Associations of PET parameters with c-MET expression and with the presence of metastasis

The intra- and inter-observer agreements of the measurements on PET/CT, expressed as ICCs, were good, with the former ranging from 0.818 for TLG to 0.907 for SUV_max_, and the latter ranging from 0.809 for TLG to 0.901 for SUV_max_ (Table 2).

**Table 2:** Intra- and Inter-observer agreements among the semi-quantitative measurements on PET/CT, expressed as intraclass correlation coefficients

Measurements	Intra-observer ICC	Inter-observer ICC
SUV_max_	0.907 (0.859–0.933)	0.901 (0.810–0.914)
SUV_peak_	0.890 (0.837–0.922)	0.884 (0.834–0.910)
MTV	0.831 (0.747–0.894)	0.841 (0.732–0.896)
TLG	0.818 (0.733–0.887)	0.809 (0.728–0.857)

Note. *SUV* standardized uptake value, *Max* maximum, *MTV* metabolic tumor volume, *TLG* total lesion glycolysis, *ICC* intraclass correlation coefficient

The mean SUVmax and SUV_peak_ of the primary gastric cancers were 8.8 (SD, 6.40) and 7.2 (SD, 5.22); the mean MTV was 57.4 mL (SD, 54.39), and the mean TLG was 328.2 g (SD, 417.44). The SUV_max_ and SUV_mean_ values were not different between c-MET-positive and negative cancers (*P* = 0.335 and 0.438). However, the MTV and TLG values were significantly larger in patients with c-MET-positive cancers compared with those with c-MET-negative cancers (*P* = 0.034 and 0.039). None of these PET parameters was significantly different between patients with locoregional tumors and those with distant metastases (Table 3).

**Table 3:** Correlations between presence of distant metastasis and PET parameters

Variables	Locoregional	Distant metastasis	*P*-values
SUV_max_ (mean ± SD)	8.3 ± 5.60	9.3 ± 6.96	0.555
SUV_peak_ (mean ± SD)	6.8 ± 4.77	7.5 ± 5.58	0.608
MTV (ml, mean ± SD)	59.4 ± 68.72	56.0 ± 42.37	0.812
TLG (g, mean ± SD)	349.4 ± 514.37	313.0 ± 339.19	0.742

Note. *SD* standard deviation, *PET* positron emission tomography, *SUV* standardized uptake value, *Max* maximum, *MTV* metabolic tumor volume, *TLG* total lesion glycolysis

### Survival outcomes in relation to parameters on PET according to c-MET expression status

In the whole patient group, SUV_max_, SUV_peak_, MTV, and TLG were all significantly correlated with patient PFS (HR = 1.065, 1.079, 1.005, and 1.001, respectively; *P* = 0.004, 0.005, 0.031, and 0.031, respectively; Table 3). SUV_max_ and SUV_peak_ correlated significantly with patient OS (HR = 1.059 and 1.071, respectively; *P* = 0.013 and 0.017, respectively; Table 4). However, the correlations between the volumetric parameters (MTV and TLG) and OS were not significant (HR = 1.005 and 1.001, respectively; *P* = 0.055 and 0.072, respectively; Table 3). Distant metastasis was significantly associated with PFS (HR = 5.009, *P* <  0.001) and OS (HR = 3.236, *P* = 0.001).

**Table 4:** Univariate linear COX regression assessing associations between parameters from ^18^F-FDG PET/CT and patient prognosis

Variables	PFS		OS	
	HR	*P*	HR	*P*
In all patients				
Gender				
Male	0.744	0.329	0.883	0.695
Female^a^	1.000		1.000	
Age (years)				
≥ 62	1.506	0.164	1.143	0.660
< 62^a^	1.000		1.000	
Pathological types				
WDA/MDA	1.252	0.447	1.103	0.749
PDA/MAC/SRC^a^	1.000		1.000	
Lauren classifications				
Intestinal	0.925	0.809	0.853	0.635
Non-intestinal^a^	1.000		1.000	
Baseline CEA (ng/ml)				
≥ 2.9	1.181	0.576	1.667	0.107
< 2.9^a^	1.000		1.000	
Baseline CA19–9 (U/ml)				
≥ 9.0	1.537	0.162	1.632	0.138
< 9.0^a^	1.000		1.000	
Baseline CA72–4 (U/ml)				
≥ 2.8	1.820	0.085	1.281	0.485
< 2.8^a^	1.000		1.000	
Findings on PET/CT				
Distant metastasis	5.009	< 0.001	3.236	0.001
Locoregional^a^	1.000		1.000	
SUV_max_	1.065	0.004	1.059	0.013
SUV_peak_	1.079	0.005	1.071	0.017
MTV	1.005	0.031	1.005	0.055
TLG	1.001	0.031	1.001	0.072
In c-Met positive patients				
SUV_max_	1.054	0.037	1.063	0.013
SUV_peak_	1.068	0.034	1.079	0.012
MTV	1.009	0.013	1.015	0.001
TLG	1.001	0.012	1.002	0.001
In c-Met negative patients				
SUV_max_	1.055	0.188	1.013	0.775
SUV_peak_	1.071	0.161	1.015	0.799
MTV	1.002	0.631	0.999	0.788
TLG	1.000	0.897	0.999	0.478

Note. *CA19–9* carbohydrate antigen 19–9, *CA72–4* carbohydrate antigen 72–4, *CEA* carcinoembryonic antigen, *WDA* well differentiated adenocarcinoma, *MDA* moderate differentiated adenocarcinoma, *PDA* poorly differentiated adenocarcinoma, *SRC* signet-ring cell carcinoma, *MAC* mucinous adenocarcinoma, *PET/CT* positron emission tomography/computed tomography, *SUV* standardized uptake value, *Max* maximum, *MTV* metabolic tumor volume, *TLG* total lesion glycolysis, *PFS* progression free survival, *OS* overall survival, *HR* hazards ratio. ^a^, reference group

When stratifying the patients according to c-MET-expression status, univariate analysis showed that in patients with c-MET-positive cancers, SUV_max_, SUV_peak_, MTV, and TLG all correlated significantly with PFS (HR = 1.054, 1.068, 1.009 and 1.001, respectively; *P* = 0.037, 0.034, 0.013 and 0.012, respectively), and OS (HR = 1.063, 1.079, 1.015 and 1.002, respectively; *P* = 0.013, 0.012, 0.001 and 0.001, respectively; Table 4). However, in patients with c-MET-negative cancers, neither PFS nor OS correlated with any of the semi-quantitative PET parameters (Table 4). In multivariate analysis, as shown in Table 5, SUV_max_ and SUV_peak_ did not correlate with patients’ outcomes, neither with PFS nor with OS. However, MTV and TLG were independent factors in predicting the patients’ OS (HR = 1.009 and 1.003, respectively; *P* = 0.024 and 0.021, respectively) after correction for distant metastasis; however, these associations were not significant for PFS (HR = 1.005 and 1.001, respectively; *P* = 0.274 and 0.350, respectively).

**Table 5:** Multivariate linear COX regression assessing associations between parameters from ^18^F-FDG PET/CT and prognosis in patients with positive c-Met expression

Variables	PFS^b^		PFS^c^		OS^b^		OS^c^	
	HR	*P*	HR	*P*	HR	*P*	HR	*P*
Findings on PET/CT								
Distant metastasis	5.620	< 0.001	5.650	< 0.001	3.026	0.002	3.151	0.002
Locoregional^a^	1.000		1.000		1.000		1.000	
SUV_max_	0.819	0.582	0.788	0.510	1.306	0.530	1.268	0.486
SUV_peak_	1.313	0.558	1.372	0.498	0.727	0.552	0.761	0.537
MTV	1.005	0.274			1.009	0.024		
TLG			1.001	0.350			1.003	0.021

Note. *PET/CT* positron emission tomography/computed tomography, *SUV* standardized uptake value, *Max* maximum, *MTV* metabolic tumor volume, *TLG* total lesion glycolysis, *PFS* progression free survival, *OS* overall survival, *HR* hazards ratio. ^a^, reference group. ^b^ This model used MTV, not TLG. ^c^ This model used TLG, not MTV

## Discussion

In this study, we investigated the prognostic values of semi-quantitative parameters on ^18^F-FDG PET/CT in patients with advanced gastric cancer according to their expression status of c-MET. The volumetric parameters of gastric cancer in situ*,* in terms of MTV and TLG, correlated significantly with positivity of c-MET expression. In addition, significant associations between volumetric parameters and the patients’ survival outcomes were identified in patients with c-MET-positive tumors, but not in patients with c-MET-negative tumors. Furthermore, the volumetric parameters, including MTV and TLG, had prognostic value in predicting the survival outcomes of patients with c-MET-positive gastric cancers.

PET/CT has the advantage of quantitatively assessing tumor metabolism using various semi-quantitative parameters, among which the SUV_max_ from a single pixel anywhere within the tumor is the most commonly used. More novel and robust is the SUV_peak_, which represents the highest mean SUV from a fixed spherical VOI centered over the highest metabolic part of the tumor. However, these parameters are two dimensional, and cannot accurately reflect the whole metabolic activity of the tumor. In contrast, volume-based parameters, such as MTV and TLG, evaluate global volume and metabolism. Previous studies have demonstrated that MTV and TLG had excellent sensitivity and specificity to predict survival outcomes of patients with cancer [17, 18]. In the present study, we found that volumetric MTV and TLG were superior to SUV_max_ and SUV_peak_ to predict the survival of patients with gastric cancer. These results were consistent with those from a study by Park et al. [5].

The activation of the c-Met/HGF pathway plays an important role in the tumorigenesis of gastric cancer [19]. Overexpression of c-MET has been proven to indicate poor prognosis in patients with gastric cancer [7, 8]. In the current study, among patients with c-MET positive gastric cancer, those who had higher tumor volumetric parameters presented a significantly shorter OS than those with lower volumetric parameters. However, this association was negative in patients with c-MET-negative gastric cancers. This suggested that the volumetric parameters on PET/CT might serve as a tool to stratify patients with gastric cancer for potential c-MET targeting therapy. The results may provide useful indications to clinicians, given that many clinical trials on c-MET targeting agent are ongoing [10, 12]. However, the volumetric parameters were not independently associated with PFS in patients with c-MET-positive cancer. This might be because of the small sample of patients included in the study and that none of these patients received any c-MET targeting therapy.

In the univariate analyses, both SUV_max_ and SUV_peak_ were significantly associated with patients’ outcomes, either with PFS or with OS, in patients with c-MET positive cancers; however, in multivariate analyses, these associations were not significant. This might be related to the collinearity of SUV_max_ and SUV_peak_ with the volumetric parameters, given that tumors with high SUV_max_ and SUV_peak_ tended to have large MTV and TLG [20, 21]. Nevertheless, the highest metabolic focus within a tumor (SUV_max_) and the metabolism of the central areas around it (SUV_peak_) may not always be associated with a large tumor burden of the primary site. This might explain why the volumetric parameters, but not SUV_max_ or SUV_peak_, were identified as independent factors to predict patients’ survival outcomes in the multivariate analyses.

There are many methods for volume segmentation, including gradient-based method, the adaptive threshold method (based on signal-to-background ratio (SBR)), and the relative or absolute SUV threshold method [22–26]. The gradient-based method is much more complex than the threshold-based method, because complicated image processing is needed, including denoising, deblurring, gradient estimation, and watershed transformation [23]. Errors can accumulate during the image-processing steps. Therefore, it is not suitable for use clinically in most PET workstations. The adaptive threshold method proposed by Daisne et al. [27] has inherent limitations for gastric cancer. First, the method relies on a calibration curve, which was validated in sphere phantoms with symmetrical volumes, homogeneous activity, and sharp demarcation. However, gastric cancers always have complex shapes and heterogeneous radioactivity levels. Second, the adaptive threshold method involves estimating the SBR assuming that the background activity is uniform. Small variations in the background activity may influence the SBR and the volume measured. The gastric wall has a wide variation of background metabolism, because of physical uptake or inflammation. Therefore, we chose the fixed-threshold method.

Gastric cancer has wide range of metabolic activity. Commonly, cancers can show low uptake of FDG, but are highly aggressive, such as poorly differentiated adenocarcinoma, signet-ring cell carcinoma, and mucinous adenocarcinoma. These tumors commonly present with low metabolic activity, even with SUV_max_ < 2.5; therefore, the threshold of ≥2.5 for delineating tumor VOI cannot be used. Therefore, we chose the 40%-SUV_max_ threshold, which has been widely used in previous studies [5, 28]. However, clinical practice indicated that the percentage threshold relies strongly on the SUV_max_ of the tumor, leading to a large variance between cancer lesions with high FDG uptakes. A method using different thresholds for tumors with different metabolic levels has being used in a previous study [5]. This method might have introduced bias to our study; however, it remains the most feasible one for use in gastric cancer.

Our study had several limitations. First, the diagnostic criteria for c-MET status were not standard. We analyzed c-MET only using protein overexpression assessed by immunohistochemical (IHC) staining. However, diagnostic criteria involving IHC have been used frequently in recent studies, because standard criteria have not been validated until now [14, 15, 19]. Second, because of the retrospective nature of this study, the studied patients did not receive c-MET targeting therapy. We cannot guarantee that the same results would be produced if patients that experienced c-MET targeting therapy were studied. Therefore, large-scale clinical trials should be conducted using patients that experienced c-Met targeting therapy to study volumetric PET/CT parameters to predict prognosis in patients with gastric cancer patients in relation to c-MET expression. Third, because of the heterogeneity of patients, the discrepancy of received treatment, and the limited sample size of population, the result could only be interpreted within this study.

## Conclusion

Gastric cancers that with positive c-MET expression had higher metabolic tumor volume and total lesion glycolysis compared with c-MET-negative gastric cancers. Volumetric parameters on ^18^F-FDG PET/CT may have a role in predicting prognosis of patients with c-MET-positive gastric cancer.

## Data Availability

The dataset used and/or analyzed in current study are available from the corresponding author on reasonable request.

## Abbreviations

18F-FDG: 2-[18F] Fluoro-2-deoxy-D-glucose
HR: Hazard ratio
ICC: Intra-class correlation coefficient
MAC: Mucinous adenocarcinoma
MDA: Moderate differentiated adenocarcinoma
MTV: Metabolic tumor volume
OS: Overall survival
PDA: Poorly differentiated adenocarcinoma
PET/CT: Positron emission tomography/computed tomography
PFS: Progression free survival
SRC: Signet-ring cell carcinoma
SUVmax: Maximum standard uptake value
SUV_peak_: Peak standard uptake value
TLG: Total lesion glycolysis
VOI: Volume of interest
WDA: Well differentiated adenocarcinoma
